# Digitale Lernmethoden im Kontext von IT-Schulungen – am Beispiel der Digitalisierung einer ERP-Fallstudie

**DOI:** 10.1365/s40702-021-00799-9

**Published:** 2021-09-29

**Authors:** Tobias Teich, Anja Brückner, Andreas Pettermann, Sebastian Wolf, Martin Trommer

**Affiliations:** 1 Vernetzte Systeme der Betriebswirtschaft, Westsächsische Hochschule Zwickau, Kornmarkt 1, 08056 Zwickau, Deutschland; 2 Fakultät Wirtschaftswissenschaften, Westsächsische Hochschule Zwickau, Kornmarkt 1, 08056 Zwickau, Deutschland

**Keywords:** Digitalisierung, Digitales Lernen, Interaktion, Lernkonzept, Wissensmanagement, Digitalization, Digital Learning, Interaction, Learning concept, Knowledge management

## Abstract

Die Corona-Pandemie machte deutlich, dass der Großteil der Unternehmen und Bildungseinrichtungen in Deutschland nicht auf die Digitalisierung alltäglicher Arbeitsprozesse eingestellt ist. Obwohl moderne Technologien – von onlinebasierten Kommunikationstools bis hin zu Künstlicher Intelligenz – mehr und mehr zur Verfügung stehen, werden diese bislang unzureichend angewandt. Wesentliche Grundlage für die Praxiswirksamkeit entsprechender Systeme ist die Verwaltung und Nutzung durch geschultes Personal. Aufgrund des IT-Fachkräftemangels fehlt dieses Personal aktuell. Zeitgleich erfolgt auch die Wissensvermittlung im Rahmen von Aus- und Weiterbildung weiterhin hauptsächlich durch Präsenzangebote. Durch das Aufeinandertreffen dieses Faktors mit dem bereits vorhandenen Fachkräftemangel entsteht eine zunehmende Divergenz zwischen wirtschaftlichen Anforderungen und bestehenden Ressourcen. Unvorhersehbare Ereignisse, beispielsweise die aktuelle Coronakrise, verstärken diese Situation weiter. Wissensvermittlung muss daher sowohl in Unternehmen als auch in Forschung an vorherrschende digitale Strukturen angepasst werden. Der vorliegende Beitrag zeigt an einem Praxisbeispiel auf Hochschulebene, wie dieser Prozess realisiert werden kann. Im Rahmen der Umsetzung wurden bestehende Vorgehensmodelle für die Konzipierung von digitalen Schulungen anhand einer SAP-Schulung umgesetzt und erweitert. Durch eine nachgelagerte Evaluation durch Lehrkräfte und Studierende, konnten wesentliche Erkenntnisse zur Funktionalität und Weiterentwicklung digitaler Schulungsangebote gewonnen werden. Prospektiv müssen diese Ergebnisse auf diverse IT-Schulungen übertragen werden, um den Kontext New Learning zu stärken und dem Fachkräftemangel nachhaltig entgegenzuwirken.

## Abstract (en)

The Corona-Pandemic showed that the majority of companies and educational institutions in Germany are not prepared for the digitization of everyday work processes. Despite available modern technology like online communication software, big data or artificial intelligence they do not use it in operation. Crucial for successful Implementation, Administration and Usage of different IT systems is trained personnel. The current lack of skilled staff in most of the companies causes a lack of personnel for IT systems. While on the other hand further education is mostly held in In-house trainings in presence. During the Covid-19-pandemic most of these trainings had to be replaced by online courses. Additionally increased by unforseen events, like the current pandemic, the divergence between economic requirements and actual resources evolves to a serious problem. That is why qualification systems need to fit the available digital structures. The following article describes a best practice way for IT-schooling at university level. The main goals are to show a practicable way to use an actual approach model for e‑learning and the combination of different online tools to develope an integrated digital SAP training experience. A feedback afterwards from the participants was useful to understand and evaluate the developed course and gave important information about functionality and user experience for upcoming courses. In the future, these results need to be adopted into different IT-trainings to strengthen the new learning context and to support the compensation for the lack of qualified personnel.

## Hintergrund – Status Quo Digitalisierung in Lehre und Unternehmenspraxis

Die Digitalisierung in der Aus- und Weiterbildung ist seit Jahren Thema in Forschung und Praxis. Zugleich war der Großteil der Unternehmen bisher nicht hinreichend auf die digitale Schulungsangebote vorbereitet. Eine Studie des Bundesverband Informationswirtschaft, Telekommunikation und neue Medien e. V. (Bitkom) (2018) zeigte, dass 57 % der Betriebe keine zentrale Strategie zur Generierung von digitalen Kompetenzen unter den Mitarbeitenden besitzen. Dies betrifft vor allem kleine und mittelständische Unternehmen. Weitere Forschungsergebnisse der Expertenkommission Forschung und Innovation machten deutlich, dass auch auf Ebene der Hochschulen lediglich 13,6 % der untersuchten Stichprobe eine ausgearbeitete Digitalisierungsstrategie besaßen (Gilch et al. 2019). Mit Beginn der Corona-Pandemie gewann dieses Defizit vermehrt an Relevanz. Sowohl Bildungseinrichtungen als auch Unternehmen waren mehr denn je gezwungen, notwendige Inhalte digital zu vermitteln, um Weiterbildung und Wirtschaftlichkeit fortlaufend zu gewährleisten. Damit einhergehend stieg im Laufe des letzten Jahres auch die Bedeutung von alternativen Lernkonzepten deutlich an. Während Virtual Classrooms und Webinare im Vorjahr von 79 % der Unternehmen als wichtig erachtet wurden, sind es im aktuellen Untersuchungszeitraum 2020/21 mit 97 % deutlich mehr (mmb Institut 2021). Zeitgleich fehlen Fachkräfte, die IT-Kompetenzen im Unternehmen einsetzen und digitale Weiterbildung vorantreiben können. Obwohl die Anzahl an Neueinstellungen während der Coronakrise zunahm, fehlen in rund sieben von zehn Unternehmen IT-Spezialisten (Bitkom 2020). Durch die digitale Transformation bedarf es neben Experten jedoch auch digitale Kompetenzen in fachfremden Berufen und Branchen. In diesem Zusammenhang nimmt auch die Qualifizierung an Hochschulen einen immer höheren Stellenwert ein (Kirchgeorg et al. 2018). Dieses Potenzial stellt, am Beispiel der Digitalisierung einer SAP-Fallstudie, das Kernthema des vorliegenden Beitrags dar. Dabei steht neben Einflussfaktoren auf die digitale Schulung auch die Umsetzung in die Praxis eine entscheidende Rolle. Durch die Anwendung eines strukturierten Vorgehensmodells sowie dem anschließenden Transfer in die Lehre, konnten umfassende Potenziale und Fallstricke bei der Digitalisierung von IT-Schulungen abgeleitet werden.

## Transformation von Präsenz zu Digital

Unabhängig von der akuten Relevanz digitaler Lernmethoden, haben sich Organisation und Inhalte von Arbeit in den vergangenen Jahren rasant verändert. Die zunehmende Adaption von „New Work“ erfordert auch die Transformation von Weiterbildungs- und Lernmethoden. In diesem Zusammenhang steht das Konzept „New Learning“, welches die Potenzial- und Selbstentfaltung des Lernenden in den Mittelpunkt stellt. Im Vergleich zum Präsenzunterricht mit klassischen Rollenkonzepten – bestehend aus Lehrenden und Lernenden – sollen die Lerneinheiten Autonomie und Individualität gewährleisten. Zugleich baut „New Learning“ auf der Idee eines gemeinschaftlichen Lernumfeldes auf, welches nur als soziales System im Austausch mit einer Gruppe funktioniert (Graf et al. 2019). Die Transformation von analogen Inhalten in ein digitales Lernumfeld ist somit singulär nicht ausreichend, um den aktuellen Ansprüchen der Arbeitswelt gerecht zu werden. Eine wesentliche Forschungsfrage daher besteht darin, welche Faktoren den Lernenden beeinflussen und so in einen digitalen Schulungskontext übertragen werden müssen, um adäquate oder sogar bessere Lernergebnisse zu erzielen.

### Einflussfaktoren auf die Erreichung von Lernzielen im digitalen Schulungsumfeld

Um die digitale Lehre optimal zu gestalten, werden in der Literatur diverse Kriterien als Orientierungspunkte für E‑Learning Kurse angeführt. So unterteilen Kergel und Heidkamp-Kergel (2019) ihre Checkliste für digitale Lehrangebote in fünf Dimensionen:

1. Strukturierung des Lernraums
  - Interaktionsmöglichkeiten
  - Transferkompetenz
  - Authentisches Lernen
  - Individuelles Lernen
2. Selbstgesteuertes Lernen
  - Handlungsorientierung
  - Produktionsorientierung
  - Selbstgesteuerte Ausgestaltung der Lernwege
  - Fehlerkultur
3. Dialogischer Austausch
  - Dialogizität
  - Dialogische Anerkennung
4. Bildungsmerkmal Selbstwirksamkeitserfahrung
  - Angemessene Lernherausforderungen
  - Metareflexion
  - Lernkontext
  - Transparente Strukturen
  - Lernbegleitung
5. Bildungsmerkmal explorative Neugier
  - Mitgestaltung der Lernherausforderungen
  - Einbindung intrinsischer Motivation

Hinzu kommen *konnektivistische Kriterien*, welche die kollaborative Nutzung dezentraler Wissensressourcen sowie das gezielte Angebot verschiedener Lernkanäle umfassen (Kergel und Heidkamp-Kergel 2019).

Auch in der Forschungspraxis konnten bereits diverse Ergebnisse zu Einflussfaktoren auf den Lernerfolg digitaler Weiterbildung gewonnen werden, welche die wesentlichen Kriterien des E‑Learning stützten. Eine aktuelle Studie von Krammer et al. (2020) konnte speziell förderliche und hinderliche Einflussfaktoren auf das digitale Lernen im Kontext von Covid-19 aufzeigen. Hierbei stand vor allem das subjektive Erleben der Kohorte, die aus Studierenden im Bereich Lehramt bestand, im Vordergrund. Es konnte eruiert werden, dass vor allem der Liveaustausch via Video und die Möglichkeit des Screensharings wesentlich für die Befragten waren. Als lernförderlich konnte weiterhin die Bereitstellung einer Lernplattform identifiziert werden. Entsprechende Lernmanagementsysteme dienen zur zentralen Organisation der Lerninhalte sowie als Planungsinstrument. Im deutschen Sprachraum sind vor allem die Lernarrangements moodle und Ilias verbreitet (Popplow 2018). Für optimale Lernerfolge sollte die zur Verfügung gestellte Plattform möglichst unterschiedliche Aufgabenbereiche sowie Diskussionsforen beinhalten. Die Studienergebnisse referieren zugleich Aspekte, die sich aus Sicht der Studierenden negativ auf den Lernerfolg auswirken. Hierzu gehörten unter anderem ein zu hoher Workload bzw. eine falsche Einschätzung des Arbeitsaufwandes (Krammer et al. 2020). Eine bundesweite Studie zum digitalen Sommersemester 2020 bestätigt diese These. Demnach besuchte fast die Hälfte der befragten Studierenden weniger Lehrveranstaltungen als in vergleichbaren Präsenzsemestern. Als häufigster Grund wurde dabei von 42,1 % eine höhere Arbeitsbelastung im digitalen Semester benannt (Traus et al. 2020). Als weiterer hinderlicher Faktor wird sowohl in der Studie nach Krammer et al. (2020) als auch in der Studie nach Traus et al. (2020) eine mangelnde technische Ausstattung angegeben. Beispielsweise zeigte sich in der Stichprobe an der Universität Essen-Duisburg, dass über 20 % der Studierenden unzureichend mit technischen Mitteln ausgestattet sind (Traus et al. 2020). Es ist zu folgern, dass Digitalisierung zwar im Alltag mehr und mehr zum Thema wird, die gegebenen Voraussetzungen jedoch nicht immer für umfangreiche Onlinekurse ausreichen.

### Relevanz menschlicher Interaktion in digitalen Lernumgebungen

Wie bereits herausgestellt, basiert der Erfolg des Lernprozesses unter anderem auf Interaktion und Gruppendynamik. Das digitale Lernen stellt hierbei eine Sonderform dar, da die Lernenden nicht im reellen Raum miteinander kommunizieren und interagieren können. Studien zeigen, dass rund 53 % der Lernenden der Meinung sind, dass Online-Lehre soziale Kontakte unter denen Lernenden zu mindestens teilweise behindert (BMBF 2018). Setzt man diese Ergebnisse in Bezug zum „New Learning“ Modell, ist die Integration der sozialen Gemeinschaft im digitalen Lernumfeld jedoch unabdingbar.

Um eine intensive Interaktion zwischen Teilnehmern zu gewährleisten, sollten Gruppen in Onlineschulungen nicht zu groß sein. Zugleich sind Zweiergruppen in der Regel nicht ausreichend, um eine hinreichende Diskussionsgrundlage zu schaffen. Studien zeigten, dass das Maximallimit für Kleingruppen bei fünf Teilnehmern liegen sollte (Qiu et al. 2014). Lehrende sollten bereits den Aufbau der Lernplattform so gestalten, dass Studierenden ausreichende Interaktionselemente zur Verfügung stehen, wobei sowohl die Interaktion zwischen den Studierenden per se als auch die Interaktion zwischen den Studierenden und Dozenten im Fokus stehen muss. Durch eine Vielzahl von Interaktionsmöglichkeiten wird das Gruppengefühl nachweislich gestärkt (Luo et al. 2017). Im Vergleich zu regulären Semestern entfiel während der Corona-Pandemie der Austausch zwischen Studierenden in Lehrveranstaltungen sowie Lerngruppen. Erhebungen aus den Niederlanden zeigten, dass für 30 % der Studierenden die Möglichkeit der Kontaktaufnahme während der E‑Learning Einheit extrem wichtig ist. Weitere 29 % der Studienteilnehmer empfanden diese Möglichkeit als sehr wichtig (Naddeo et al. 2021).

### Identifikation transformationsrelevanter Elemente bei digitalen Schulungsdesigns

**Abb. 1:**
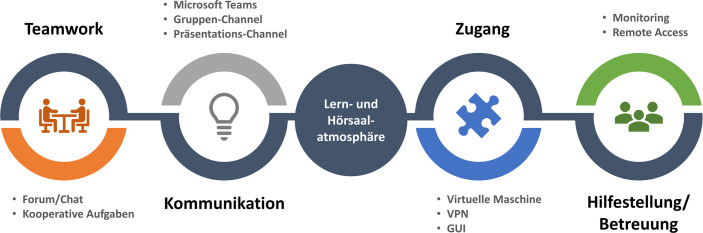
Elemente zur Transformation in ein digitales Kursformat

Bildung ist ein Gut welches durch digitale Medien und Vernetzung mehr Menschen als jemals zuvor zur Verfügung steht. Häufig fehlen dabei jedoch wichtige Aspekte (siehe 2.1), die einen dauerhaften Lerneffekt oder eine positive Lernatmosphäre gewährleisten. Im Vordergrund steht oft lediglich die Weitergabe der Informationen. In der Entwicklung der vorliegenden SAP Best Practice Schulung wurden bereits in der Entwicklungsphase weitere Einflussfaktoren, wie die Kommunikation zwischen Teilnehmenden und Betreuenden sowie die Gruppendynamik einbezogen. Bei der Analyse der Präsenzveranstaltungen ergaben sich fünf wesentliche Aspekte, die anschließend in den zweiwöchigen Online-Kurs zu transportieren waren. Grundlage hierfür war die Dokumentation der Kernprozesse aus den Präsenzkurse der letzten fünf Jahre sowie eine daraus folgende Ableitung und Priorisierung der Anforderungen. Die wesentlichen herangezogenen Elemente sind in Abb. 1 dargestellt.

#### Hörsaal- und Lernatmosphäre

Zunächst braucht es eine Anwendung, die eine Kommunikation zwischen Betreuenden und Teilnehmenden ermöglicht und ähnlich wie ein Hörsaal funktionieren kann. Aufgrund des vielfältigen Angebots an Kommunikationsplattformen erfolgte zunächst ein Test, mit welcher Software die benötigten Funktionen realisierbar sind. Hierzu zählten die Möglichkeiten, Präsentationen zu teilen, Sprach- und Textchat, Screensharing, Erstellung von Gruppenräumen, Kompatibilität und Verfügbarkeit. An der Westsächsischen Hochschule Zwickau (WHZ) wurden zum Ist-Stand drei Anwendungen verwendet: Big Blue Button, Zoom und Microsoft Teams. Alle drei Plattformen erfüllen im Wesentlichen die notwendigen Voraussetzungen. Die Entscheidung fiel aufgrund vorher durchgeführter Tests mit kleinen Studierendengruppen auf Microsoft Teams, da sowohl die Handhabung als auch die Performance überzeugten.

#### Zugang und Verfügbarkeit

Zugang und Verfügbarkeit stellen bei IT-Schulungen ein zentrales Problem dar. Hardware und Software, die in Hochschulen mit speziellen Lizenzen vorhanden ist, stehen den Anwendern im privaten Umfeld oft nicht zu Verfügung. Eine praktikable und effiziente Lösung wurde in der Kombination von Virtual Private Networks und Virtual Machines gefunden. In Zusammenarbeit mit der zentralen Stelle für Informationsverarbeitung an der Hochschule wurden mittels der vorhandenen Login-Daten für die Studierenden Remote-Zugänge geschaffen. Hierbei wurde darauf geachtet, dass die Software sowohl für Windows als auch für Mac OS kompatibel ist.

#### Kommunikation zwischen Betreuenden

Die Aufgaben der Betreuenden umfassen im Wesentlichen die Präsentation von Theorieinhalten und Hilfestellungen in der Praxisanwendung. In den Präsenzveranstaltungen ist es möglich, sich direkt abzustimmen und die Aufgaben untereinander zu verteilen. Dies konnte aufgrund der Corona-Regelungen nur bedingt gewährleistet werden. Da die Plätze begrenzt waren, musste ebenfalls eine onlinebasierte Kommunikation gewährleistet werden. Hierfür wurden entsprechende Betreuerchats und Räume in Microsoft Teams eingerichtet, die für die Studierenden nicht zugänglich waren und lediglich zur Abstimmung der Betreuer verwendet wurden. Dies gewährleistete kurze und schnelle Kommunikationswege.

#### Teamwork innerhalb Teilnehmergruppen

Für die Durchführung der Fallstudien wurden die Teilnehmer in Gruppen eingeteilt, welche während der Bearbeitung kooperative Aufgaben untereinander besprechen und abstimmen mussten. Die Kommunikation innerhalb der Gruppen ist nicht nur aus Bearbeitungssicht relevant, sondern auch aus Teilnehmersicht. Gruppendynamik und Zusammenarbeit fördern, wie in Punkt 2.1 beschrieben, das Lernumfeld und so die Motivation der Teilnehmer über die Zeit des Kurses.

#### Informationsbereitstellung

Während der Schulung werden Dokumente in Form von Präsentationen, PDF und Excel Dateien verteilt. Diese sollen den Kursteilnehmern zentral zur Verfügung gestellt werden. Da die Kurse wiederholt stattfinden, wurde an dieser Stelle auf das Lernplattformsystem moodle zurückgegriffen. An der Hochschule werden die Vorlesungsunterlagen ebenfalls dort zur Verfügung gestellt, so dass die Zugänge für die Kursteilnehmer bereits vorhanden sind und darauf zurückgegriffen werden konnte. Weiterhin war den Studierenden das System bekannt, was die praktische Anwendung erleichterte.

## Best Practice am Beispiel einer ERP-Fallstudie

### Ist-Stand vor Transformation

Die SAP-Ausbildung an der WHZ ist seit vielen Jahren als Modul im Master Management fest verankert. Die Veranstaltungen sind gebündelt in einem zweiwöchigen Kurs. In dieser Zeit erhalten die Teilnehmer ca. 1000 Seiten Theoriefolien und ca. 800 Seiten SAP-Klickanleitung, welche während des Kurses erklärt und in angeleiteten Übungen ausgeführt werden. Ziele des Kurses sind die Vermittlung und Verknüpfung von betriebswirtschaftlichen Grundlagenwissen mit theoretischen Grundlagen des SAP S/4HANA Systems und die anschließende Umsetzung anhand der integrierten Fallstudie (Teich und Käschel 2008; Teich et al. 2020). Die Betreuung der Kurse wird von Mitarbeitenden und Studierenden aus dem Arbeitskreis Integrierte Informationssysteme übernommen. In Präsenz werden die Kurse in einem technischen Kabinett der Hochschule durchgeführt, so dass die Studierenden einen barrierefreien Zugang zur notwendigen Hard- und Software erhalten. Die Kurse umfassen meist zwölf bis fünfzehn Personen. Diese werden in Dreiergruppen aufgeteilt, die je eine Supply-Chain (OEM, Supplier 1 und 2) abbilden. Der OEM soll am Ende ein Fahrzeug, den „Milo One“, herstellen. Hierfür werden Bauteile benötigt, die entweder extern beschafft, von einem Mitglied der Supply Chain hergestellt oder geliefert werden. Die Kommunikation der Teilnehmer untereinander ist an dabei besonders relevant. Die Supply-Chain beinhaltet feste Abläufe, für welche eine Abstimmung untereinander erforderlich ist.

### Umsetzung der Digitalisierung der Fallstudie

Mit Beginn der Corona-Pandemie bestand auch für die WHZ die akute Herausforderung, Präsenzlehre ins digitale Umfeld zu verlagern. Im Sommersemester 2021 erforderte das Infektionsgeschehen zwingend ein digitales Format, welches im vorangegangenen Semester systematisch geplant wurde. Vor Beginn des Kurses wurde allen Teilnehmern ein Testtag angeboten, dass zu Beginn des Kurses der Zugriff auf die notwendigen Systeme für alle sichergestellt war. Über die Lernplattform moodle wurden alle Dokumente, wie PDF-Dateien zur Fallstudie, den Vorträgen und den Zugangsdaten bereitgestellt. In Microsoft Teams nutzten die Betreuenden einen Gruppenraum für die Vorträge und die Beantwortung von Fragestellungen, die alle Kursteilnehmer betreffen. Diese hatten die Möglichkeit, den Video- und Sprachchat oder den Textchat zu nutzen.

Die Bearbeitung der praktischen Anteile erfolgte anschließend in Gruppenräumen mit je sechs Teilnehmern und einem festen Betreuenden. Dieser begleitete die zugeteilte Gruppe bei der Durchführung der Klickanleitung und stand bei Problemen während der Ausführung am System oder bei Fragen zur Verfügung. Bei komplexeren Herausforderungen erfolgte eine Abstimmung zwischen dem Trainerteam. Zunächst wurde mit den Teilnehmern versucht über den Sprachchat eine Lösung zu finden. War die Fehlersuche in diesem Schritt nicht erfolgreich, erfolgte ein Screensharing. War auch dieser Weg nicht hinreichend, konnte sich der Betreuende über die Bildschirmsteuerung auf den Computer des Teilnehmers zuschalten und ihn zur Lösung führen. Diese Arbeitsweise hat sich im Laufe des Prozesses in der Praxis als sehr zweckdienlich herausgestellt.

### Evaluation

**Abb. 2:**
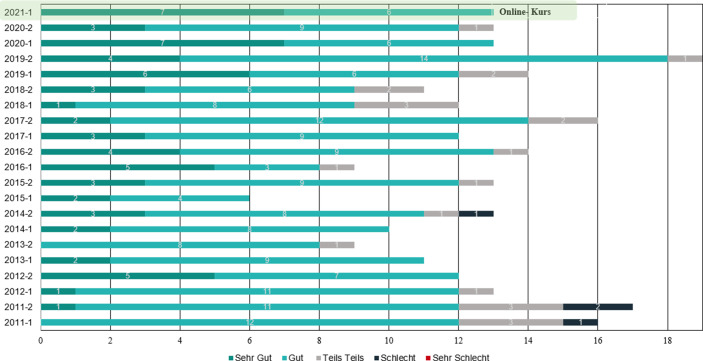
Auswertung Feedbackbogen

Im Anschluss an den Kurs wurde den Teilnehmenden über moodle ein Feedbackbogen zur Verfügung gestellt. Dieses Verfahren hat sich bereits in zurückliegenden Kursen etabliert, da die Rückmeldungen der Teilnehmenden in zukünftige einbezogen werden soll. Für den ersten digitalen Kurs 2021 erhielten wir 13 von 18 Rückmeldungen. Auch wenn diese Ergebnisse nicht als repräsentativ zu betrachten sind, liefern sie doch wertvolle Ansatzpunkte zur Entwicklung des Kurses. Die Bewertungen sind in Winter- (‑1) und Sommersemester (‑2) aufgeteilt. Abb. 2 gibt eine Übersicht über die erhaltenen Gesamtbewertungen der letzten Jahre. Aus dieser wird ersichtlich, dass sich die Bewertung des Online-Kurses mit dem vorangegangener Präsenzkurse ähnelt.

Aus den quantitativen Daten des Feedbackbogens konnten weiterhin folgende Schlüsse gezogen werden.

- Die Teilnehmenden waren mit dem Aufbau des Kurses, der Organisation sowie der Umsetzung überwiegend zufrieden (8- sehr gut, 5‑ gut).
- Die Organisation des Online-Kurses wurde als sehr positiv bewertet (9- sehr gut, 4‑ gut).
- Die Vorträge waren gut verständlich (6- sehr gut, 5‑ gut, 1‑ teils teils, 1‑ schlecht).
- Es gab ein ausgewogenes Angebot an theoretischen und praktischen Inhalten (5- sehr gut, 4‑ gut, 4‑ teils teils).
- Das Lernklima wurde als durchweg positiv empfunden (7- sehr gut, 6‑ gut).
- Die Umsetzung der onlinebasierten Schulung wurde als positiv und praktikabel bewertet (8- sehr gut, 5‑ gut).

Weiterhin wurden qualitative Daten erhoben, die in Form von Ergänzungen angegeben werden konnten. Anmerkungen gab es bezüglich entstandenen Leerlauf- und Pausenzeiten. Dies ist eine Konsequenz daraus, dass erst weitergearbeitet werden konnte, wenn alle Teilnehmenden die jeweilige Aufgabe erledigt hatten und keine Fehler mehr in den Dateneingaben vorhanden waren. Zur Kompensation sollen in Zukunft noch weitere Übungen und Zusatzmaterialien für das Selbststudium bereitgestellt werden.

Kritikpunkte sahen die Teilnehmenden beim hohen fachlichen Input der Vorträge. Aufgrund der Komplexität der Themen und der Kursdauer, stellt dieser Faktor auch in Präsenz eine Herausforderung dar. Zusätzlich ist die Verständlichkeit im Onlineformat von der Vortragsgeschwindigkeit und Mikrofonqualität abhängig, so dass hier zukünftig noch Ansatzpunkte für Verbesserungen bestehen. Eine Schwierigkeit, die fast alle Teilnehmenden als kritisch empfanden bestand darin, die Konzentration zum genauen Lesen über die Dauer des Kurses aufrecht zu halten. Zusammengefasst wurden die Strukturierung der Inhalte sowie der Kurs als Gesamtes jedoch trotz der Remote-Organisation von allen als empfehlenswert bewertet. Die gewonnenen Ergebnisse sowie das Gesamtfeedback der Teilnehmenden konnten zeigen, dass die Präsenzschulung erfolgreich in ein Onlinekonzept überführt wurde. Da sich die SAP-Fallstudie hinsichtlich der Prozesse nicht sehr von anderen komplexen IT-Schulungen unterscheidet, können diese Ergebnisse auch auf andere IT-Trainingssettings übertragen werden. Beispielsweise für Programmierschulungen, welche nicht nur im Rahmen des Informatikstudiums, sondern aufgrund der digitalen Transformation auch in betrieblichen Weiterbildungen eine zunehmende Rolle einnehmen.

## Fazit und Ausblick

Zusammenfassend konnte gezeigt werden, dass entscheidende Elemente, die von Präsenzveranstaltungen in das digitale Umfeld transformiert werden müssen vor allem im Bereich Interaktion sowie der technischen Ausstattung liegen. Die aktuellen Ergebnisse aus der Forschung zeigen, dass einerseits der dringende Bedarf an Weiterbildungen im IT-Bereich gegeben ist (Krammer et al. 2020; BMBF 2018). Andererseits kann dieser Bedarf aufgrund der Umstrukturierung von Arbeit, der hohen Anzahl der Zu-Qualifizierenden sowie wirtschaftlicher Ausnahmesituationen nicht ausschließlich in klassischen Präsenzkonzepten erfolgen. Die Transformation von Lehrinhalten in ein digitales Trainingsumfeld erfordert jedoch die Eruierung und Berücksichtigung relevanter lerndidaktischer Vorgehensweisen. Das Praxisbeispiel zeigt, dass die Transformation durchaus gelingen kann und wesentlicher Handlungsbedarf vor allem in bekannten Bereichen, wie unzureichender technischer Ausstattung oder überproportioniertem Workload, auftritt. Im Rahmen der angewandten Vorgehensweise handelte es sich jedoch um ein Pilotprojekt, welches weiterentwickelt und auf eine größere Teilnehmeranzahl sowie andere Systemschulungen transferiert werden muss. Auch wird die Zukunft mehr und mehr von digitalen Lebensräumen geprägt sein, die das digitale Lehrumfeld zur unabdingbaren Konsequenz für die Qualifizierung von Personal machen. Die im Rahmen der Corona-Pandemie notwendige Anwendung von E‑Learning sollte somit nur als ein grundlegender Impuls bei der Veränderung und Digitalisierung von Weiterbildung interpretiert werden.
